# Comparison of the area of the pharynx during wakefulness and induced sleep in patients with Obstructive Sleep Apnea (OSA)

**DOI:** 10.1590/S1808-86942012000100016

**Published:** 2015-10-20

**Authors:** Ana Célia Faria, Luis Vicente Garcia, Antonio Carlos dos Santos, Paula Rejane Beserra Diniz, Helcio Tadeu Ribeiro, Francisco Veríssimo de Mello-Filho

**Affiliations:** a Doctoral degree in medical science, Ribeirão Preto Medical School, São Paulo University. Oral and maxillofacial surgeon, Integrated Center for the Study of Facial Deformities (Centro Integrado de Estudo das Deformidades da Face or CIEDEF) Clinic Hospital, Ribeirão Preto Medical School, São Paulo University.; b Adjunct professor, Biomechanics, Medicine and Rehabilitation of the Aparelho Locomotor Department, Ribeirão Preto Medical School, São Paulo University.; c Associate professor, Internal Medicine Department, Clinic Hospital, Ribeirão Preto Medical School, São Paulo University.; d Master's degree in neuroscience, bachelor degree in biomedical informatics, doctoral student, Neuroscience Department, Ribeirão Preto Medical School, São Paulo University.; e Specialist in orthodontics, doctoral student, Ophthalmology, Otorhinolaryngology and Head & Neck Surgery Department, Ribeirão Preto Medical School, São Paulo University.; f Associate professor and head of the Integrated Center for the Study of Facial Deformities, Ophthalmology, Otorhinolaryngology and Head & Neck Surgery Department, Ribeirão Preto Medical School, São Paulo University.

**Keywords:** magnetic resonance imaging, obstructive, pharynx, sleep apnea

## Abstract

The study of obstructive sleep apnea (OSA) has received growing attention over the past years since various aspects have not been sufficiently established.

**Aim:**

To evaluate, with the use of magnetic resonance imaging (MRI), changes in the area of the pharynx during wakefulness and induced sleep in patients with OSA.

**Materials and Methods:**

A prospective study of thirty-two patients with a polysomnographic diagnosis of OSA. All patients were submitted to MR imaging in order to obtain high-definition anatomical sagittal sequences during wakefulness and during sleep induced with Propofol. An area was defined on the sagittal plane in the midline of the pharynx. This region was called pharyngeal midplane (PMP) area.

**Results:**

A significant difference in PMP area (mm^2^) was observed between wakefulness and induced sleep in each patient (*p* < 0.000001).

**Conclusion:**

The patients with OSA suffer a significant reduction of 75,5 % in the area of the pharynx during induced sleep compared to wakefulness.

## INTRODUCTION

The obstructive sleep apnea syndrome (OSAS) has been studied in greater depth to investigate several relevant aspects of its physiology pathology and therapy that are not sufficiently clear. A clear understanding of upper airway structures and function in OSAS patients is needed to further understand the pathogenesis of this disorder and to select the most appropriate therapy.

With this in mind, the site of obstruction in upper airways has been studied using several approaches, such as the physical examination, nasopharyngolaryngoscopy, and image methods – cephalometrics, computed tomography (CT), and magnetic resonance (MR) imaging^1^. A limitation of methods that evaluate OSAS is that they are done with patients awake, often in a sitting or standing position, and thus do not faithfully reproduce the upper airway morphology during sleep. It is, therefore, not always possible when patients are awake to identify even a simple narrowing of upper airways as a cause of the events involved in airway occlusion during sleep.

MR imaging has been used in OSAS patients to locate oropharyngeal obstructions and abnormalities^2^, to study upper airways and adjacent soft tissues^3^, ^4^, ^5^, ^6^, ^7^, ^8^, to assess fat deposits in airways^9^, ^10^, to find anatomical factors in airways at risk for OSAS^11^, and to study possible anatomical differences in the airways of males and females^12^. A functional assessment of patients in decubitus and sleeping is needed to locate any obstruction, as the morphology of the airways varies when patients are awake or sleeping^13^.

Polysomnography is done while patients are asleep. It is able to detect disease severity, and whether it is of central or obstructive origin; this method, however, is unable to detect an obstruction site in upper airways. Thus, morphological studies of upper airways are routinely carried out with patients awake in an attempt to locate the obstruction site. This difference may be the cause of difficulties to identify anatomical obstruction sites, and to indicate the most appropriate procedure for the treatment of OSAS.

There have been few published studies using MR imaging to assess OSAS patients in the sleeping state^14^, ^15^, ^16^, ^17^, ^18^. The emphasis in these studies has been either to compare the morphology of OSAS patients with that of normal controls or to establish a relationship between disease severity and obstruction sites.

We found no studies on differences in pharyngeal area (mm^2^) in OSAS patients measured in wakefulness and sleep.

### Objective

The purpose of this study was to assess changes in the pharyngeal area of OSAS patients in wakefulness and induced sleep by using MRI.

## MATERIALS AND METHODS

The sample comprised 32 patients aged from 18 to 62 years, diagnosed with OSAS. The inclusion criterion was OSAS demonstrated by polysomnography as defined by an apnea/hypopnea index (AHI) over five events per hour. Exclusion criteria were use of medication and neuromuscular diseases. The clinical history and physical examination based on a standard protocol at our unit were part of the procedure for all patients in the sample. The institutional review board approved this study on human beings (protocol number 6245/2006).

### Polysomnography

Polysomnography was done in the Sleep Laboratory of the Clinical Neurophysiology Section of the hospital; it consisted of an entire night recording. Patients were admitted from 7 to 10 p.m. and discharged at 7 a.m. on the next morning.

At the sleep laboratory, patients answered a standard questionnaire before and after polysomnograph recordings. A Digital BioLogic Polysomnograph – Poliwin 2000 software was used, and the recording time was at least 5 hours. The study variables were grouped into an internationally proposed standard format for polysomnography.

### Magnetic Resonance Imaging

All patients were referred to the Image Unit of the hospital for MR imaging, which was done on a Magneton Vision 1.5 Tesla superconducting device (Siemens, Erlangen, Germany) with a 25 milliTesla gradient magnetic field, and an emit and receive radiofrequency head coil with a circular polarized component.

The image acquisition protocol included anatomical high-definition sagittal T1-weighted images followed by a dynamic study with T1-weighted fast ecogradient sequences with patients awake.

Next, sleep was induced by endovenous Propofol (3 to 5 mg per Kg) by an anesthesiologist, after which images were again acquired as soon as patients were asleep.

DICOM images were transferred to an auxiliary workstation.

### Analysis of images

Measurements were taken using the Display software (McGill University, Montreal, Canada).

**Figure 1:**
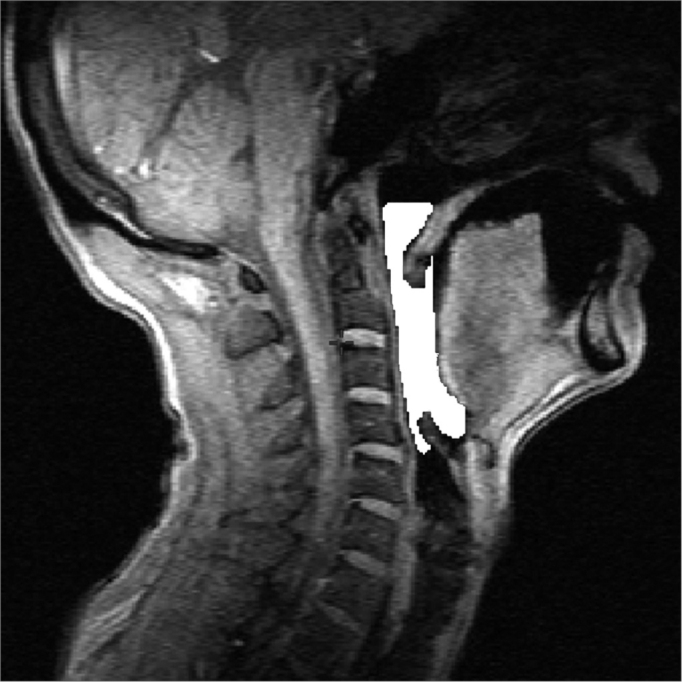
Image showing the PMP area defined superiorly by a line passing along the hard palate to the posterior pharyngeal wall, inferiorly by the back of the vallecula, posteriorly as the posterior pharyngeal wall, and anteriorly as the base of tongue and the soft palate.

The area that was assessed was only the air space; hard or soft tissues were not included. This area was defined on the sagittal plane of the pharyngeal midplane, superiorly by a line passing along the hard palate to the posterior pharyngeal wall, inferiorly by the back of the vallecula, posteriorly as the posterior pharyngeal wall, and anteriorly as the base of tongue and the soft palate (Figure 1). This region was named the pharyngeal midplane (PMP) area. The PMP area was measured pixel by pixel in sagittal images using the abovementioned software, which yields simultaneous views of three orthogonal planes and makes it possible to accurately define the anatomy. It generates a binary file from which the area may be calculated.

### Analysis of the PMP area of patients awake and under induced sleep

The first step was an analysis on the sagittal plane to compare the area of interest in the same patient while awake and in induced sleep. Two sequences of the same spatial location were used, one section with the patient awake and one with the patient in induced sleep. The PMP was drawn by thresholding to measure the maximum size (mm^2^). Only the maximum PMP measure was used, as the shortest measure, generally seen when patients were in induced sleep, was collapse of the area. This procedure avoided including other areas that were not of interest for this study, and reduced interference of the partial volume as much as possible. The software calculated the areas automatically and inserted the numbers into spreadsheets for statistical comparisons.

## RESULTS

**Chart 1:**
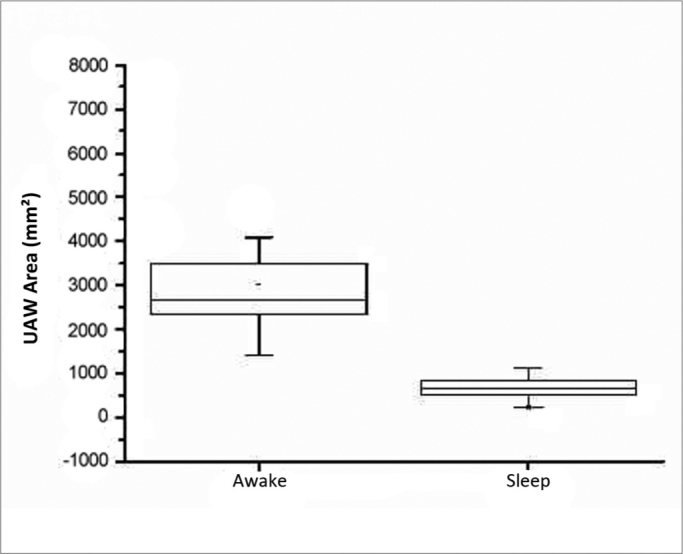
Distribution of area measurements (mm^2^) of upper airways in wakefulness and induced sleep.

**Chart 2:**
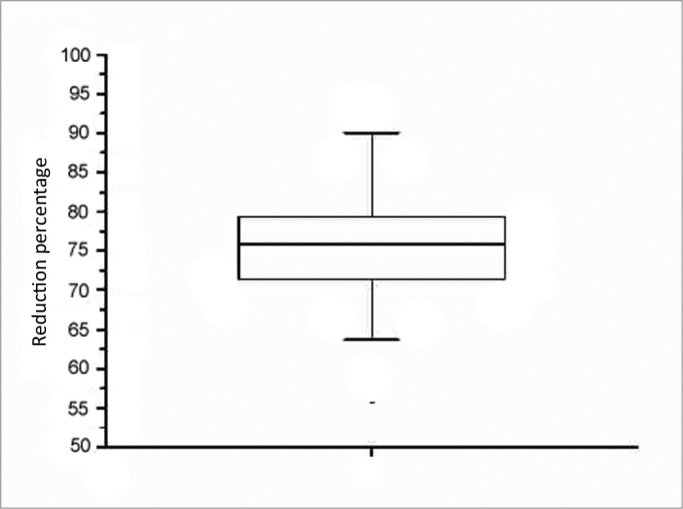
Graphic representation of the percentage reduction of the upper airway area during induced sleep.

There were 23 male and nine female patients in the sample of 32 OSAS subjects. The mean AHI was 29.55 events per hour of sleep; the mean BMI was 26.97 (Table 1). Table 2 shows the PMP measures (mm^2^) and wakefulness and induced sleep for each patient, the differences among these measurements, and the percentage reduction of the pharyngeal area during induced sleep. A comparison of values gathered from patients awake and in induced sleep yielded a statistically significant difference (*p*< 0.000001). The upper airway values in wakefulness and induced sleep are presented graphically based on its distribution in mm^2^ (Chart 1). The mean reduction of the pharyngeal area during induced sleep was 75.55% (SD 7.44) (Chart 2).

**Table 1:** Data on 32 patients – age group (years), AHI (events/ hour of sleep) and BMI (Kg/cm^2^).

Patients	Age	AHI	BMI
1	43	18.2	19.92
2	39	47.2	25.77
3	56	8.1	29.55
4	54	26.2	26.58
5	62	56.3	26.45
6	23	12	23.24
7	37	22	34.97
8	49	9.2	26.4
9	34	61.5	26
10	45	41	25.51
11	44	42	24.62
12	43	45	22.4
13	38	5.7	24.34
14	38	16	31.46
15	44	9	20.27
16	15	29.3	21.08
17	44	15.7	21.45
18	48	34	23.94
19	30.7	39	24.42
20	60	43.1	26.64
21	26	42	31.1
22	52	6	24.05
23	44	47	43.8
24	42	32.5	31.12
25	43	13.77	34.08
26	50	8	29.65
27	32	25.3	25
28	57	40.3	26.27
29	25	24.55	22.31
30	43	47	36.65
31	18	15.7	20.24
32 mean	39 41.18	63.1 29.55	33.8 26.97

IAH – Apnea/Hypopnea Index.

IMC – Body Mass Index.

**Table 2:** Measures of the PMP area (mm^2^) based on MR images of 32 patients awake and during induced sleep, the differences among measures, and percentage of reduction of the pharyngeal area.

Patients	Wakefulness	Sleep	Differences among measures	Percentage reduction or area (%)
1	6044.7	1116.8	5927.9	81.5
2	3945.9	1008.9	2937	74.4
3	2389.8	433.9	1.955.9	81.8
4	5307.9	531.9	4.776.0	90.0
5	3022.3	862.5	2159.8	71.5
6	3909	702.6	3206.4	82.0
7	3166.1	793.3	2372.8	74.9
8	2237.5	536.4	1701.1	76.0
9	4086.1	985.6	3100.5	75.9
10	2077.2	578.6	1498.6	72.1
11	3787.8	486.1	3301.7	87.2
12	2443.7	566	1877.7	76.8
13	2972	1078.5	1893.5	63.7
14	3629.7	635.2	2994.5	82.5
15	1786.1	236.3	1549.8	86.8
16	2670.1	549.8	2120.3	79.4
17	2389.8	545.3	1844.5	77.2
18	3453.6	846.2	2607.4	75.5
19	2371.9	721.4	1650.5	69.6
20	2249.7	487.8	1761.8	78.3
21	3500.3	882.3	2618	74.8
22	2034.1	433.9	1600.2	78.7
23	1405.2	415	989.2	70.5
24	3202	664.8	2537.2	79.2
25	1728.6	729.5	999.1	57.8
26	2350.3	651.4	1698.9	72.3
27	3489.5	817.6	2671.9	76.6
28	2407.4	476.2	1931.2	80.2
29	2368.3	689	1678.3	70.9
30	2806.8	849.0	1957.8	69.7
31	2573.1	665.7	1907.4	74.1
32	3388	1502.2	1885.8	55.7

mm^2^ – square millimeters.

PMP – pharyngeal midplane.

## DISCUSSION

Because surgery for the treatment of OSAS results in permanent anatomical changes and do not require patient compliance after the procedure is undertaken, studies of the preoperative anatomy of upper airways may significantly help select the ideal technique^19^. Therefore, this study presents a standard objective approach for preoperative evaluation in which the reduction of the upper airway area may be defined numerically while the patient is asleep.

A few studies have shown that OSAS patients generally present a reduced airway space, which may lead to occlusion during sleep^15^. These studies, however, have not objectively measure this reduction.

We found in this study that although the upper airways are wide enough for air to pass when patients are awake, they undergo a major reduction in the area (75%) during sleep, resulting in obstruction (Table 2 and Chart 2). This is an important finding, as we found no similar result in the medical literature.

Suto et al.^14^ (1993) published a study of 15 OSAS patients in which the upper airways were assessed subjectively by MR with patients awake and in induced sleep. Their results indicated significant anatomical changes in the upper airways of 87% of their sample when evaluated during induced sleep. Our results, however, show in a plain and measurable way that the PMP area was significantly reduced in 100% of the study sample during sleep.

Such a significant change in the upper airways appears to determine the severity of OSAS, as Suto & Inoue^15^ have reported; these authors concluded that multiple obstruction sites in the pharynx are associated with severe apnea.

The size of the airway in the pharynx is determined by an interaction between neural regulation of dilatory muscle activity and structure. Pharyngeal collapse during sleep in OSAS patients could therefore be cause by abnormalities of these factors^20^.

Anatomical factors, such as craniofacial abnormalities (retrognathism, micrognathism, maxillary atresia, overbite), an abnormal soft palate, macroglossia, hypertrophic tonsils, and nasal block, may contribute to the onset of apnea^21^. There are, however, OSAS patients that have no obvious anatomical changes. Our results showed that the method we described may identify and measure area reductions in these cases.

Abbey et al.^22^ (1989) suggested that OSAS patients do not always present a narrow pharynx while awake, but that they may have variable sites of narrowing in the upper airways that collapse during sleep. Thus, especially in patients with no obvious anatomical changes, function (coordination of pharyngeal dilatory muscles) becomes important. A morphological assessment of upper airways is essential during wakefulness and sleep as the pharyngeal dilatory muscle activity differs in these two states.

Our results demonstrate a significant reduction (*p*< 0.000001) of the air space during induced sleep – with collapse sites within the PMP area – in the entire sample. These anatomical changes do not appear and cannot be diagnosed when patients are tested awake. Thus, MR imaging in wakefulness and induced sleep was able to accurately identify and measure upper airway changes.

## CONCLUSION

The mean pharyngeal area of OSAS patients in our sample was significantly reduced – by 75.55% – during induced sleep compared to wakefulness.
